# Bioinformatics analysis of common key genes and pathways of intracranial, abdominal, and thoracic aneurysms

**DOI:** 10.1186/s12872-020-01838-x

**Published:** 2021-01-06

**Authors:** Siwei Bi, Ruiqi Liu, Linfeng He, Jingyi Li, Jun Gu

**Affiliations:** 1 West China School of Medicine, Sichuan University, Chengdu, Sichuan People’s Republic of China; 2 Department of Burn and Plastic Surgery, West China Hospital, Sichuan University, Chengdu, Sichuan People’s Republic of China; 3 Department of Cardiovascular Surgery, West China Hospital, Sichuan University, Chengdu, Sichuan People’s Republic of China

**Keywords:** Aneurysm, Bioinformatics analysis, Weighted gene co expression network analysis

## Abstract

**Background:**

Aneurysm is a severe and fatal disease. This study aims to comprehensively identify the highly conservative co-expression modules and hub genes in the abdominal aortic aneurysm (AAA), thoracic aortic aneurysm (TAA) and intracranial aneurysm (ICA) and facilitate the discovery of pathogenesis for aneurysm.

**Methods:**

GSE57691, GSE122897, and GSE5180 microarray datasets were downloaded from the Gene Expression Omnibus database. We selected highly conservative modules using weighted gene co‑expression network analysis before performing the Gene Ontology, Kyoto Encyclopedia of Genes and Genomes pathway and Reactome enrichment analysis. The protein–protein interaction (PPI) network and the miRNA-hub genes network were constructed. Furtherly, we validated the preservation of hub genes in three other datasets.

**Results:**

Two modules with 193 genes and 159 genes were identified as well preserved in AAA, TAA, and ICA. The enrichment analysis identified that these genes were involved in several biological processes such as positive regulation of cytosolic calcium ion concentration, hemostasis, and regulation of secretion by cells. Ten highly connected PPI networks were constructed, and 55 hub genes were identified. In the miRNA-hub genes network, CCR7 was the most connected gene, followed by TNF and CXCR4. The most connected miRNAs were hsa-mir-26b-5p and hsa-mir-335-5p. The hub gene module was proved to be preserved in all three datasets.

**Conclusions:**

Our study highlighted and validated two highly conservative co-expression modules and miRNA-hub genes network in three kinds of aneurysms, which may promote understanding of the aneurysm and provide potential therapeutic targets and biomarkers of aneurysm.

## Background

Aneurysms are permanent dilation of the arteries, secondary to pathologic changes of arterial walls. They can occur at any site of arteries and may lead to serious outcomes such as rupture and dissection [1, 2], which are related to high mortality. Abdominal aortic aneurysm (AAA), thoracic aortic aneurysm (TAA) and intracranial aneurysm (ICA) are three common kinds of aneurysms [3–5]. Intriguingly, previous studies have shown the co-occurrences of any two of them [6–10] at different levels. Furthermore, Kim et al. noted the familial association of aortic aneurysm and ICA [11], indicating the possible common genetic predisposition underlying the aortic and cerebral aneurysm.

Recently, whole-genome linkage studies have identified some genomic loci serving as common genetic risk factors. For example, 4q32-34 and 19q for AAAs and IAs, 3p24-25 and 5q for TAAs and IAs, and 18q11 and 15q21 for all three kinds of aneurysm [12, 13]. Besides, an increasing number of gene-expression regulators and key genes were identified in different aneurysms [14–16]. With the development of gene sequencing techniques, there are an increasing number of studies focusing on the expression profile of artery aneurysm [17–19]. However, there are no studies focusing on identifying how gene expression profile preserved in these three kinds of aneurysms.

Therefore, three gene expression microarray datasets in the Gene Expression Omnibus (GEO) were included in the present study. Weighted gene co‑expression network analysis (WGCNA) was used to construct a co-expression network, and the highly preserved expression modules with genes were selected. Subsequently, we performed a comprehensive functional enrichment analysis of the selected modules. The protein–protein interaction (PPI) was conducted with the identification of highly connected genes and their targeted miRNAs. In the present study, the in‑depth analysis may increase the knowledge into the pathogenesis of aneurysm, along with key genes and therapeutic targets for the human aneurysm.

## Methods

### Microarray data download and preprocessing

We downloaded GSE57691, GSE122897, and GSE5180 microarray data from NCBI GEO (http://www.ncbi.nlm.nih.gov/geo/). After excluding the control datasets, there are 49 datasets from samples obtained from patients with AAA in GSE57691, 44 intracranial aneurysm datasets from the intracranial cortical artery samples in GSE122897, and 12 thoracic ascending aortic aneurysm datasets from the patients with tricuspid aortic valve. The platforms for GSE57691, GSE122897, and GSE5180 are Illumina HumanHT-12 V4.0 expression beadchip, Illumina HiSeq 2500 (Homo sapiens), and Affymetrix Human Genome U133A Array, respectively. Raw data of these datasets were preprocessed using R software (version 4.0.3, https://www.R-project.org/). Specifically, we implemented the preprocessing pipeline in limma [20] package for GSE57691. For GSE122897, the transcript abundance was filtered at a CPM of 0.5 and the trimmed mean of M values (TMM) was implemented to normalize the raw data with the help of the edgeR [21] Bioconductor package. The uploaded GSE5180 data has normalized already. The annotation for the probes and clinical traits information were downloaded using GEOquery [22] package. After excluding the probes that are unable to be annotated, we combined the probes annotated with the same genes using the median method.

### WGCNA construction and module selection process

WGCNA [23] package was required for the co-expression network construction. Firstly, we selected common genes in the three datasets and chose the forty percent genes with the most variance in the GSE57691. After evaluating the correlations between the three datasets using the verbose Scatterplot function, we calculated the soft threshold value based on a scale-free topology criterion in GSE57691 (scale-free R2 = 0.9). The weighted adjacency matrix was constructed using the soft-thresholding power. Relationships between one gene and all the other genes in the analysis were incorporated, and the adjacency matrix was transformed into the topological matrix (TOM). Subsequently, a hierarchical clustering analysis [24] of genes was performed using 1‑TOM as the distance measure. To acquire a small number of large modules, modules were detected using a dynamic tree cut algorithm with a minimum module size of 50 and a minimum cut height of 0.99. Furthermore, module preservation between the two datasets was measured using the specific function of the WGCNA [23] software package. In general, the higher the value of "Zsummary.pres" the more preserved the module is between data sets: 5 < Z < 10 indicates moderate preservation, while Z > 10 indicates high preservation. We selected the modules with high preservation in both GSE122897 and GSE5180.

### Gene Ontology (GO), Kyoto Encyclopedia of Genes and Genomes (KEGG) pathway and Reactome enrichment analysis

GO [25] analysis was a popular method to elucidate potential biological processes (BP), molecular functions (MF), and cellular components (CC) associated with the genes. KEGG pathway database contains information about the mechanism of networking between molecules or genes. It complements the majority of the existing molecular biology databases with further information, including information on the individual gene [26]. Biological pathways enriched in the selected modules were also interrogated with the Reactome pathway database. We performed the enrichment analysis in Metascape (http://metascape.org/), a gene annotation, and analysis resource [27]. The threshold for P-value was set at 0.01 and the minimum enrichment score was 1.5. The heatmap of enriched terms was plotted and the genes that belong to the same enriched ontology term were shown in a Circos plot [28].

### PPI network and hub gene identification and validation

PPI enrichment analysis was carried out using the following databases: BioGRID [29], InWeb_IM [30], and OmniPath [31] in Metascape (http://metascape.org/). Molecular Complex Detection (MCODE) [32] was used to screen the densely connected network with the default parameters in the whole PPI network. For each MCODE component, pathway and process enrichment analysis has been applied. The three functional description terms with best-scoring p-value have been retained. The genes within each component were selected as hub genes.

### The construction of the miRNA-hub genes network

The interactions of miRNA-hub genes were predicted using the miRNet [33] web-based platform. By uploading the list of gens IDs of interest, users can map genes to their microRNAs (miRNAs) according to the miRTarBase [34] v8.0, TarBase [35] v8.0 and miRecords [36]. The results were presented as each row representing the interaction between one miRNA and its target and visualized in Cytoscape [37]. 3.7.2 software. The interactions between two genes were acquired from the STRING [38] database. We implemented the yFiles Layout Algorithms app (“https://www.yworks.com/products/yfiles-layout-algorithms-for-cytoscape)” to construct a circular layout. In the network, a node represents a gene or a miRNA; the undirected link between two nodes is an edge.

### The validation of the hub gene module

Taking the GSE57691 as the reference dataset and the hub genes as a module, we validated the hub gene module by examining the reproductivity (or preservation) in three independent test datasets (GSE13353:ICA, GSE7084:AAA, GSE98278:AA) with the method illustrated in Langfelder et al.’s work [39]. The download and normalization methods for the validation datasets were as previously stated. The module preservation measurements can be influenced by several factors such as the size of the network and the modules etc. Hence, 200 permutation tests were applied to assess the significance of the preservation statistics. Taking advantage of aggregating multiple preservation statistics into summary preservation statistics, all of the density and connectivity based preservation measures were summarized using three composite Z statistics Z_density_, Z_connectivity_ and Z_summary_ [40, 41]. In case that modules are defined as clusters, the in group proportion (IGP) statistic, a benchmark statistic for module preservation assessment [42], was also calculated.

## Results

### Identification of highly preserved WGCNA modules

**Fig. 1:**
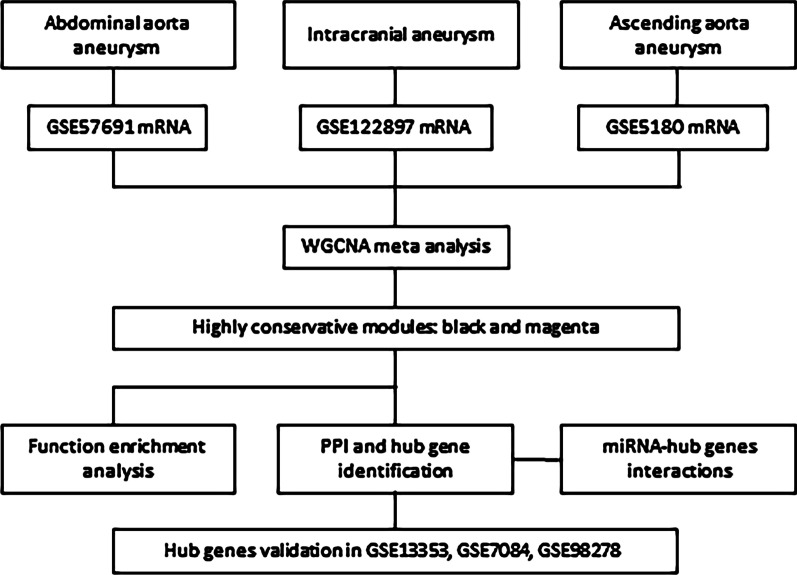
Flow chart diagram for analysis process. WGCNA: weighted gene co‑expression network analysis; PPI: protein–protein interaction

**Fig. 2:**
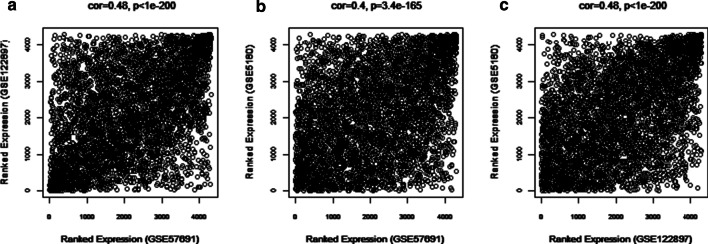
Expression correlation analysis of genes in three datasets. *Cor* correlation coefficient

**Fig. 3:**
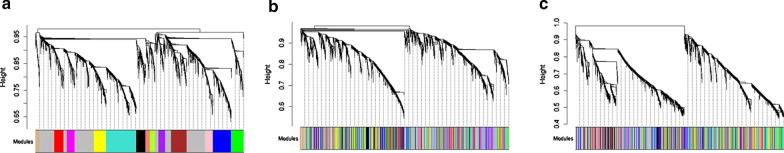
Dendrogram and clustering for identification of gene co‑expression modules between **a** GSE57691, **b** GSE122897, and **c** GSE5180

As illustrated in Fig. 1, our study attempted to identify co‑expression modules with high preservation of artery aneurysm from different locations: abdominal (GSE57691), intracranial (GSE122897) and thoracic (GSE5180) using WGCNA. We selected 4306 genes with the aforementioned method and the weighted adjacency matrix was constructed using six as the soft-thresholding power (Additional file 1). Then, we assessed the correlation of average gene expression between the three datasets (Fig. 2). The correlation coefficients of any of the two datasets were all statistically significant which suggests that these datasets were suitable for further analysis. Then, we set the parameter “deepSplit” = 0 to achieve a small number of large modules (Additional file 2). In the GSE57691 dataset, 14 modules are identified as the training set and were reconstructed as a validation set in the GSE122897 and GSE122897 datasets (Fig. 3). These modules are illustrated in the branches of the dendrogram with unique colors. In 2 of the 14 modules, only the black and magenta modules were well preserved, with Z‑scores > 10 in both validation datasets (Table 1). Thus, the black (193 genes) and magenta (159 genes) modules were selected for further analysis.

**Table 1:** The preservation between GSE57691, GSE122897 and GSE5180

	Preservation scores of GSE57691 and GSE122897		Preservation scores of GSE57691 and GSE5180	
	Module size	Zsummary.pres	Module size	Zsummary.pres
Black	193	18.90972	193	21.62663
Blue	379	1.56026	379	5.612991
Brown	324	14.72513	324	7.96055
Gold	100	0.211093	100	2.695959
Green	245	0.547156	245	7.127068
Greenyellow	106	2.573385	106	0.799292
Grey	400	5.571804	400	3.64588
Magenta	159	23.46621	159	13.45767
Pink	159	0.39723	159	2.606454
Purple	141	16.21788	141	6.828419
Red	196	− 0.02071	196	6.77718
Salmon	83	22.78504	83	8.145203
Tan	84	5.575885	84	5.30432
Turquoise	400	0.43602	400	4.791903
Yellow	253	1.426967	253	14.08676

### Enrichment analysis of the genes in the highly conserved modules

**Fig. 4:**
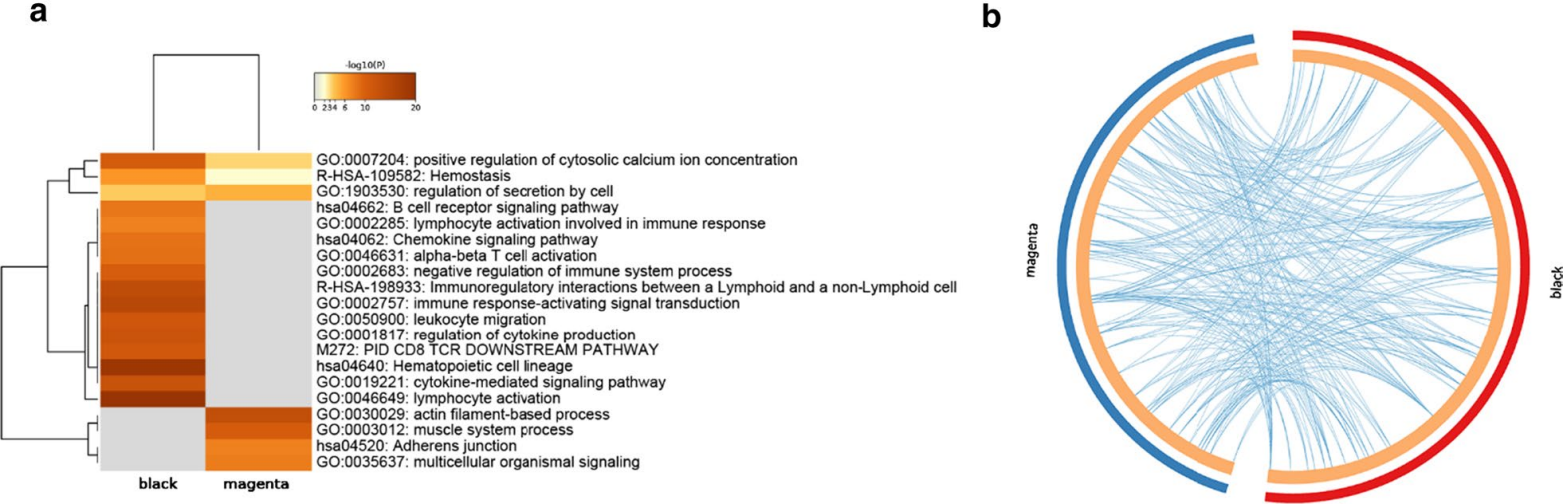
**a** Function enrichment analysis for genes in magenta and black modules. The top 20 enriched terms were illustrated and color by *p* values. **b** Overlap of enriched terms between the genes in magenta and black modules. Blue curves link genes that belong to the same enriched ontology term. The inner circle represents gene lists, where hits are arranged along the arc

To improve our understanding of the biological information of the two modules in artery aneurysm, we performed comprehensive functional enrichment analysis in various databases (Fig. 4a). Up to 100 enriched terms can be accessed in Additional file 3. Terms such as positive regulation of cytosolic calcium ion concentration, hemostasis, and regulation of secretion by cells were enriched in both datasets. Meanwhile, the results for black module were the immune-related terms mostly such as lymphocyte activation and immune response-activating signal transduction and for magenta module, actin filament-based process and muscle system process were enriched. As shown in Fig. 4b, there were considerable genes that belong to the same enriched ontology term in magenta and black modules.

### Construction of PPI network and hub gene identification

**Fig. 5:**
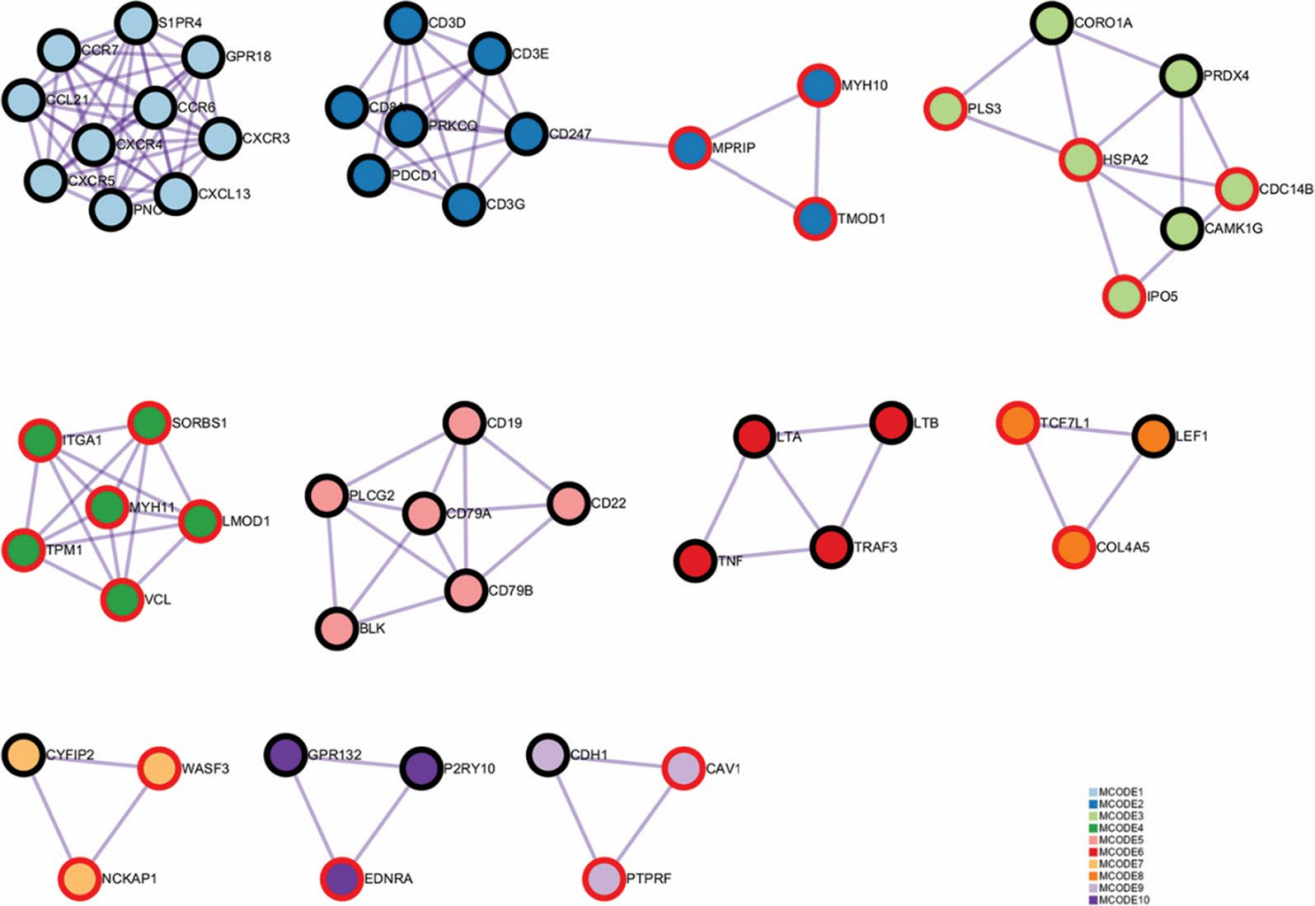
Protein–protein interaction network and MCODE components identified in the gene lists. MCODE: Molecular Complex Detection. The edge of node represents the module they belong, i.e., red for the magenta module and black for black module

We downloaded the resultant network from the Metascape and processed it with Cytoscape [37]. 3.7.2 software. The MCODE analysis indicated 10 highly connected networks, in which 35 genes were from black module and 20 genes were from magenta module (Fig. 5). The top three enriched functional description terms with best-scoring p-value were tabulated in Table 2. The analysis did not yield any results for MCODE 3. Taken all the identified networks together, positive regulation of cytosolic calcium ion concentration, lymphocyte activation, and regulation of cytosolic calcium ion concentration were identified. All 55 genes were treated as hub genes.

**Table 2:** Three functional description terms with best-scoring p-value of each MCODE identified network

Network	Annotations #1		Annotations #2		Annotations #3	
MCODE ALL	GO:0007204	positive regulation of cytosolic calcium ion concentration	GO:0046649	lymphocyte activation	GO:0051480	regulation of cytosolic calcium ion concentration
MCODE 1	R-HSA-373076	Class A/1 (Rhodopsin-like receptors)	R-HSA-418594	G alpha (i) signalling events	R-HSA-500792	GPCR ligand binding
MCODE 2	hsa04660	T cell receptor signaling pathway	M88	PID CD8 TCR PATHWAY	M272	PID CD8 TCR DOWNSTREAM PATHWAY
MCODE 4	R-HSA-445355	Smooth Muscle Contraction	R-HSA-397014	Muscle contraction	GO:0006936	muscle contraction
MCODE 5	R-HSA-983695	Antigen activates B Cell Receptor (BCR) leading to generation of second messengers	R-HSA-983705	Signaling by the B Cell Receptor (BCR)	GO:0050853	B cell receptor signaling pathway
MCODE 6	hsa04064	NF-kappa B signaling pathway	R-HSA-5668541	TNFR2 non-canonical NF-kB pathway	R-HSA-5676594	TNF receptor superfamily (TNFSF) members mediating non-canonical NF-kB pathway
MCODE 7	R-HSA-5663213	RHO GTPases Activate WASPs and WAVEs	R-HSA-2029482	Regulation of actin dynamics for phagocytic cup formation	R-HSA-9664407	Parasite infection
MCODE 8	hsa05200	Pathways in cancer				
MCODE 10	R-HSA-416476	G alpha (q) signalling events	R-HSA-373076	Class A/1 (Rhodopsin-like receptors)	R-HSA-500792	GPCR ligand binding

### Construction of miRNA-hub genes network

**Fig. 6:**
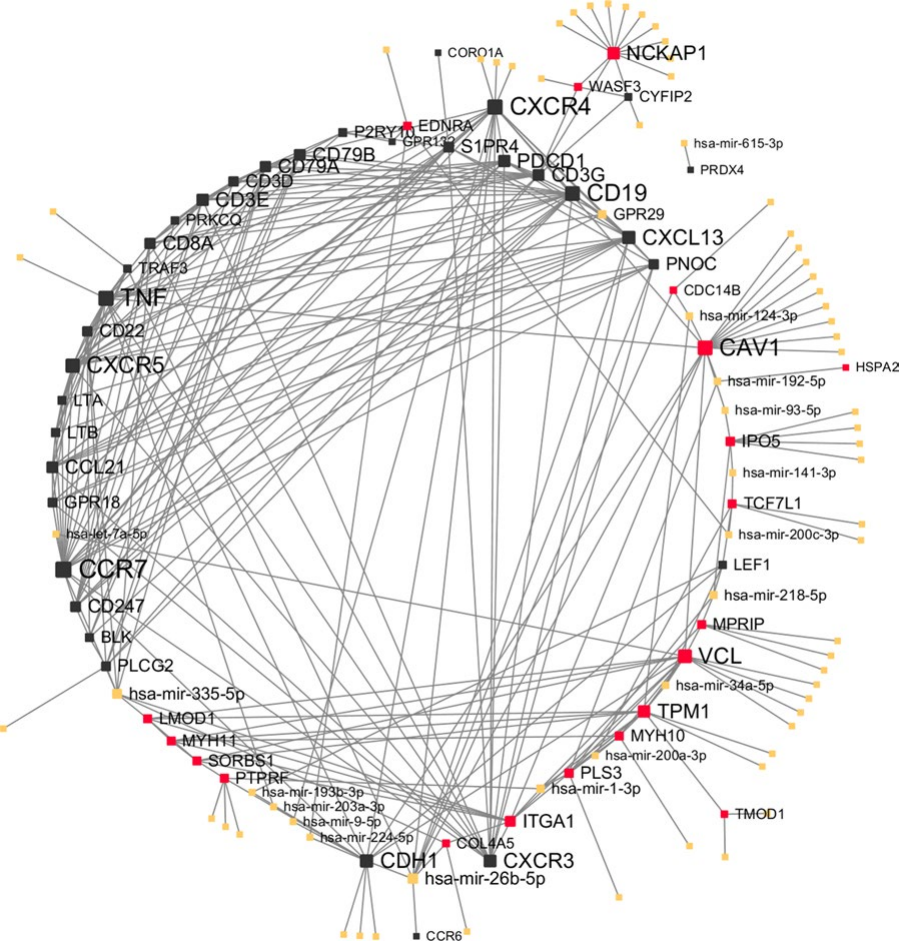
miRNAs and hub genes interactions illustration. The genes in the black module and magenta module are colored in black and red, respectively. The size of each nodes represents the number of interactions with other nodes. All the miRNAs were in yellow

We uploaded the hub gene lists in the black and magenta modules to the miRNet website, and filtered the results so that only miRNA-gene interactions verified in at least two databases would be shown. The interactions are illustrated in Fig. 6. The genes in the black and magenta module and miRNAs were in black, red and yellow respectively. Notably, the CCR7 was the most connected gene (21 degrees) while TNF and CXCR4 interacted with 19 other nodes. The most connected miRNAs were hsa-mir-26b-5p and hsa-mir-335-5p with 9 and 8 degrees.

### Validation of the hub genes module

**Fig. 7:**
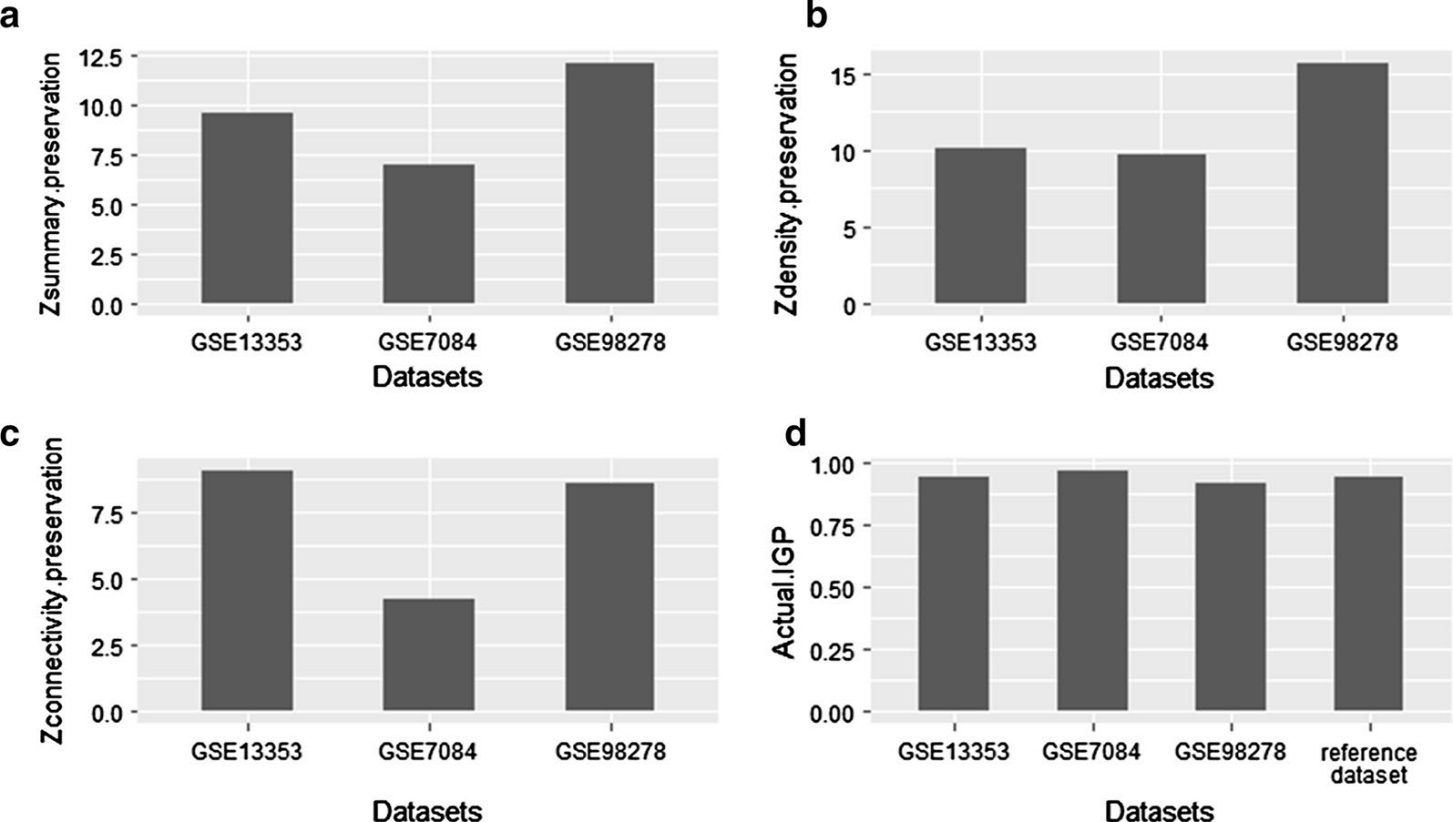
Quantitative evaluation of the similarities among the networks. **a** the composite preservation statistic Z_summary_. The hub gene module in the reference dataset is highly preserved in the GSE98278 (Z_summary_ > 10) and moderately preserved in the other two datasets. **b**, **c** the density and connectivity statistics, respectively. **d** Results of the in group proportion (IGP) analysis shows that the hub gene module is equally preserved in all network

**Fig. 8:**
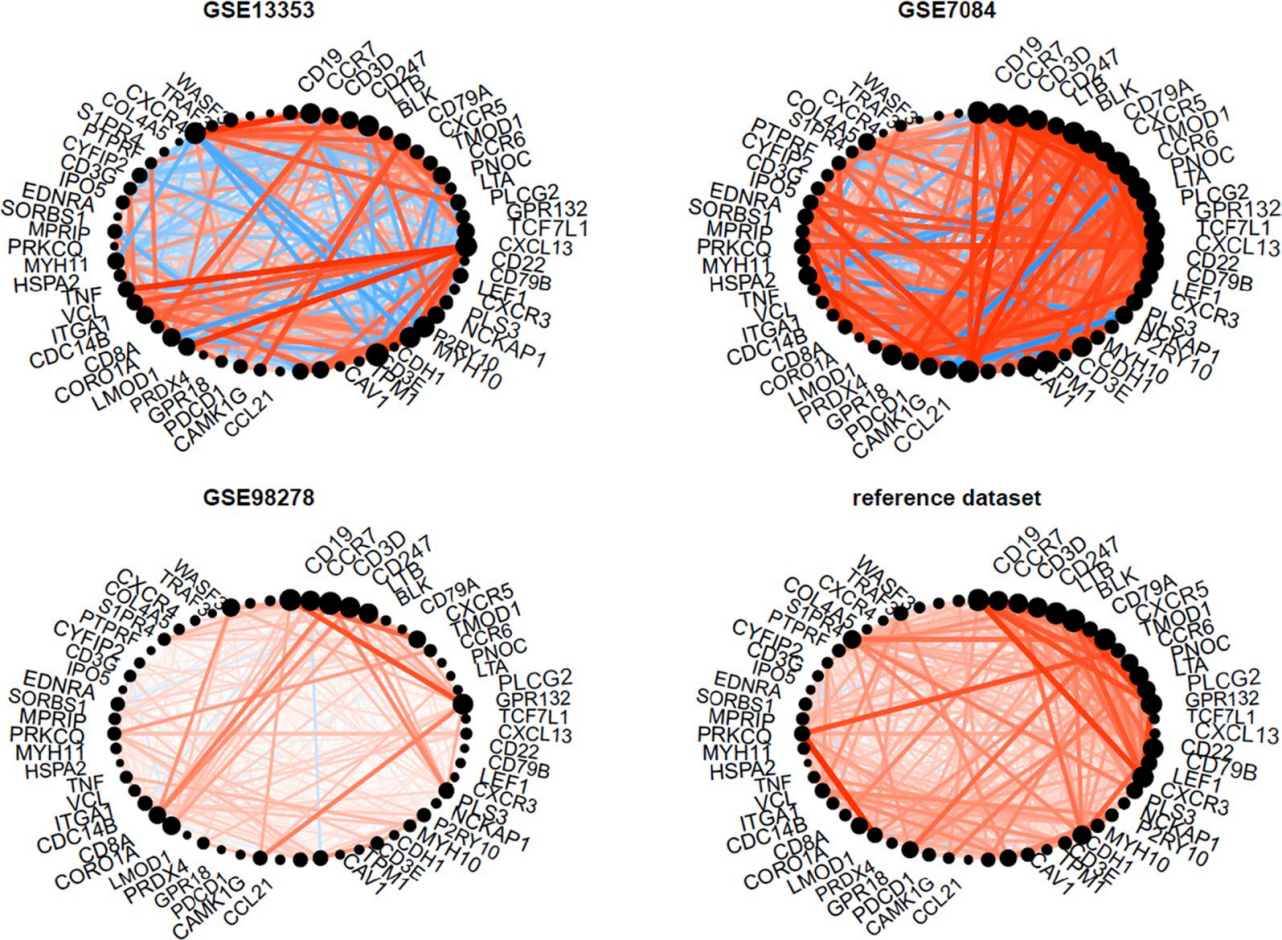
Network plot of the hub gene module in three validation datasets. Positive correlations are represented by red lines, while negative correlations are represented by blue lines. Correlation strength is represented by thickness and color saturation of the line. Intramodular hub genes are represented by larger points and their names

Another 3 gene expression datasets: GSE13353, GSE7084, GSE98278 were downloaded from the GEO database. The detailed information and normalization methods for each dataset used in this study were shown in Additional file 4. For all three datasets, the hub gene module showed consistent conservation as proved by the four main statistics we focused (Fig. 7). GSE98278 achieved the highest value in Z_summary_ preservation (12.13) while the GSE7084 showed a moderate but still a good Z_summary_ preservation score (6.97). The full analyse results for all module preservation statistics were attached in Additional file 5. Moreover, we plotted the hub gene module network in these three validation datasets (Fig. 8). Note the similarity between the three validation datasets and the reference dataset. CCR7 is consistently highlighted in all datasets.

## Discussion

One of the key features of pathological changes in aneurysm is inflammatory infiltration and enzymatic destruction of the elastic lamellae and extracellular matrix (ECM) proteins [43]. Plasma Membrane Calcium ATPase 4 (PMCA4) plays a role in transporting calcium from the cytosol to the extracellular and it is also known to be expressed in aortic tissue [44]. Kinza et al. [45] have demonstrated that the expression of PMCA4 could be reduced in human primary aortic endothelial cells during inflammation which enhanced the proteins related to ECM remodeling. Especially, another study conducted by Alexander et al. [46] reported that mitochondrial calcium uptake 2 (Micu2) is an intermitochondrial membrane protein functioning to reduce the amount of calcium coming into the mitochondrial matrix [47]. They showed that naive Micu2^−/−^ mice could develop abdominal aortic aneurysms with spontaneous rupture with modest blood pressure elevation and concluded Micu2 is crucial in protecting the abdominal aorta. In our analysis, the term “positive regulation of cytosolic calcium ion concentration” is underlined in our enrichment analysis for the highly conserved modules and also the hub genes. More thorough knowledge of how the potential regulators including PCMA4 and Micu2 take part in the cytosol calcium concentration regulation during artery aneurysm progression will support the development of therapeutic strategies.

Interestingly, the hub genes in MCODE 6 were predicted to participate in the NF-kappa B pathway with tumor necrosis factor (TNF) as a key player. The other three genes are Lymphotoxin-α and -β (LTA and LTB), and TNF receptor-associated factor 3 (TRAF3). LTA, also called TNF-β, is produced by lymphocytes and is involved in various cellular activities such as promoting lymphoid tissue development and inflammatory and immune responses [48]. In an earlier study conducted by Magne et al. in the human myeloma cell line OH-2., TNF mediates NF-kappa B activation via both TNF receptors, whereas LTA does so only via TNF-R1. Similarly, LTB, together with its receptor, is also a critical role in the immune system development, response and activation of the pro-inflammatory NF-kappa B pathway [49]. In vascular smooth muscle cells (VSMCs), the LTB receptor has been demonstrated to participate in atherosclerosis protection via artery tertiary lymphoid organs [50]. A better understanding of the role of lymphotoxin may help to guide the development of a new therapeutic strategy to treat aneurysm.

Various miRNAs have been identified as key regulators that modulate the pathogenesis of aneurysm [51, 52]. In our results, miR-26b-5p and miR-335-5p were highlighted. Studies have revealed that miR-26b-5p plays a negative regulatory role in the angiogenesis process of hepatocellular carcinoma. Recently, Changwu et al. reported miR‑26b‑5p regulated the transforming growth factor β (TGFβ)/Smad4 signaling pathway to modulate hypoxia‑induced phenotypic switching of VSMCs. On the other hand, harnessing the results of a prior study [53], Bei et al. [54] designed a novel device that would determination of the severity of AAA by detecting miRNA-335-5p. However, little is known about the specific regulation network of these two miRNAs in the aneurysm. Our study provides novel insights for future studies that focus on researching how miRNAs take part in aneurysms.

There are some limitations in the present study. Firstly, our results are based on pure public data with unavoidable biases, such as age and gender differences. Additionally, further in-vivo and in-vitro experimental exploration and validation for the identified genes and modules are required.

## Conclusions

To conclude, the present study comprehensively identified the highly conservative co-expression modules and hub genes in three kinds of aneurysms. The potential biological function of the modules, hub genes and the miRNA-hub gene interactions may possess important clinical implications for the treatment and diagnosis of aneurysm.

## Supplementary information

**Additional file 1.** Construction of weighted adjacency matrix. 4306 genes were included for the construction of the weighted adjacency matrix and the authors used six as the soft-thresholding power.

**Additional file 2.** Gene clustering and different deepsplit method. The parameter “deepSplit” was set to 0 to achieve a small number of large modules.

**Additional file 3.** The 100 enriched terms for the genes in magenta and black modules.

**Additional file 4**.The datasets used in the project for module preservation identification.

**Additional file 5**. Full analyse results for all module preservation statistics

## Data Availability

The datasets used and/or analysed during the current study are available from the corresponding author on reasonable request.

## Abbreviations

TGFβ: Transforming growth factor β
VSMCs: Vascular smooth muscle cells
TNF: Tumor necrosis factor
LTA and LTB: Lymphotoxin-α and -β
TRAF3: TNF receptor-associated factor 3
Micu2: Mitochondrial calcium uptake 2
ECM: Extracellular matrix
PMCA4: Plasma membrane calcium ATPase 4
PPI: Protein–protein interaction
miRNAs: MicroRNAs
MCODE: Molecular complex detection
BP: Biological processes
MF: Molecular functions
CC: Cellular components
GO: Gene ontology
KEGG: Kyoto encyclopedia of genes and genomes
TOM: Topological matrix
TMM: Trimmed mean of M values
GEO: Gene expression omnibus
WGCNA: Weighted gene co‑expression network analysis
AAA: Abdominal aortic aneurysm
TAA: Thoracic aortic aneurysm
ICA: Intracranial aneurysm
