# Supplementary figures and images for: Bioinformatics analysis of common key genes and pathways of intracranial, abdominal, and thoracic aneurysms
Source: BMC Cardiovasc Disord. 2021 Jan 6;21:14. doi: 10.1186/s12872-020-01838-x (PMC7788746; doi:10.1186/s12872-020-01838-x)

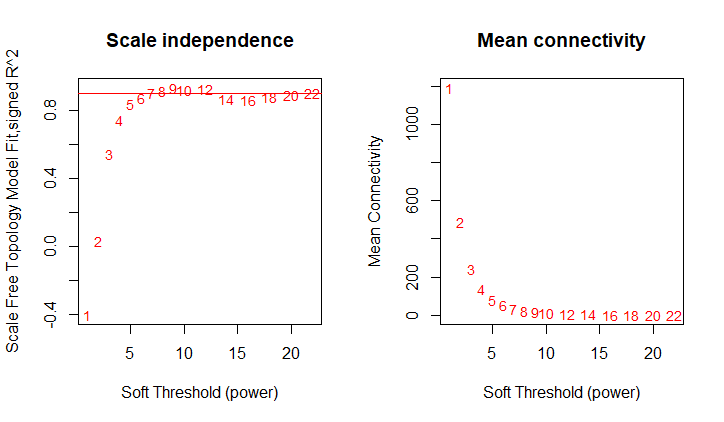

Supplement: Supplementary file 1 — Additional file 1. Construction of weighted adjacency matrix. 4306 genes were included for the construction of the weighted adjacency matrix and the authors used six as the soft-thresholding power. [file 12872_2020_1838_MOESM1_ESM.tiff]

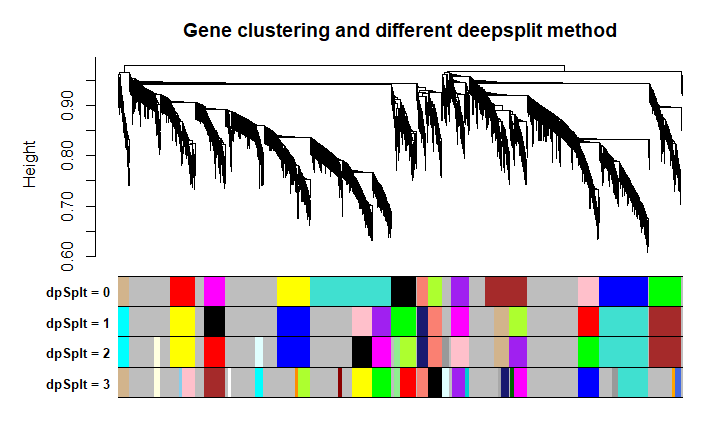

Supplement: Supplementary file 2 — Additional file 2. Gene clustering and different deepsplit method. The parameter “deepSplit” was set to 0 to achieve a small number of large modules. [file 12872_2020_1838_MOESM2_ESM.tiff]

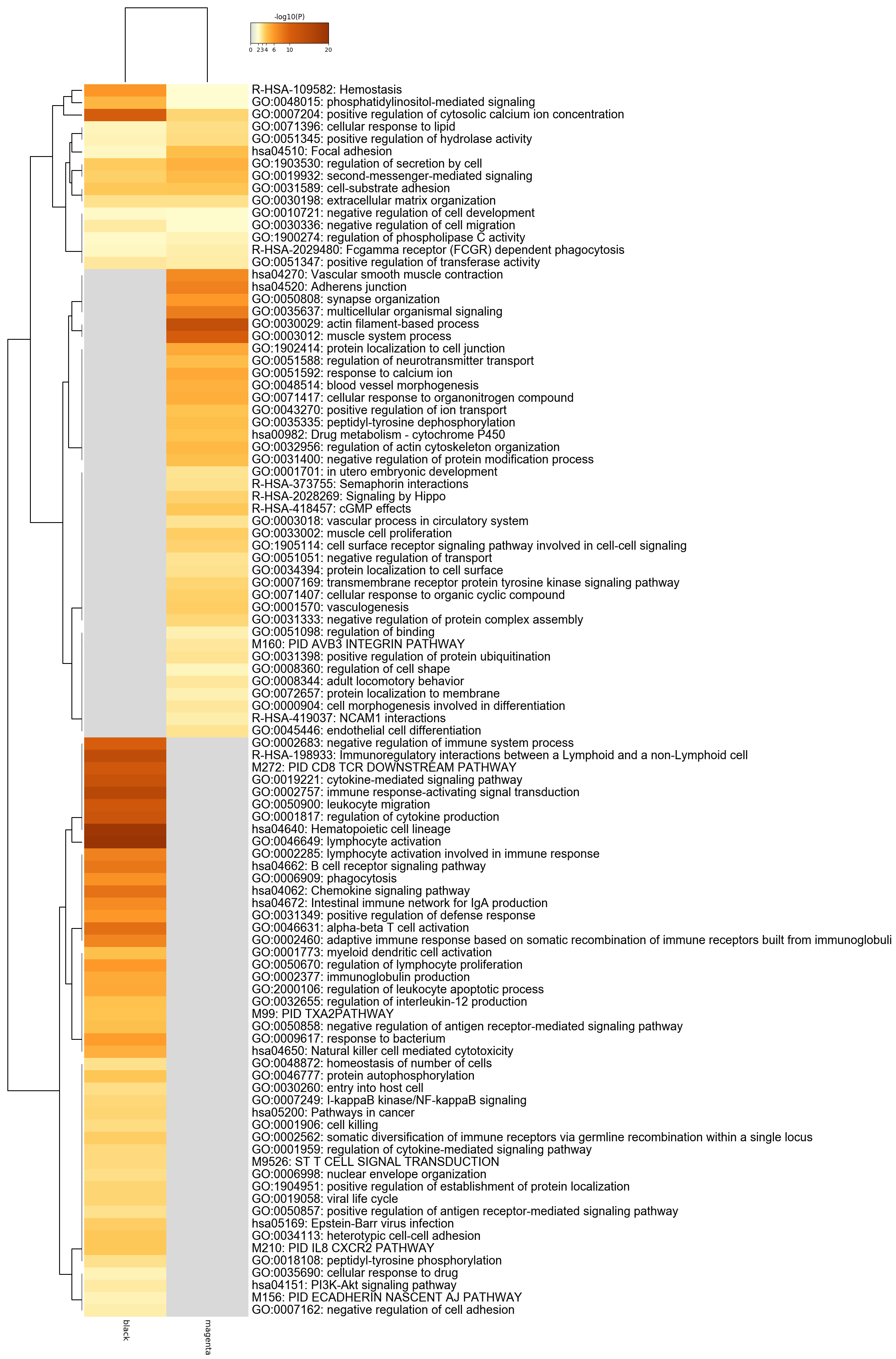

Supplement: Supplementary file 3 — Additional file 3. The 100 enriched terms for the genes in magenta and black modules. [file 12872_2020_1838_MOESM3_ESM.png]
